# Oxymatrine Improves Intestinal Epithelial Barrier Function Involving NF-κB-Mediated Signaling Pathway in CCl_4_-Induced Cirrhotic Rats

**DOI:** 10.1371/journal.pone.0106082

**Published:** 2014-08-29

**Authors:** Jian-Bo Wen, Fang-Qing Zhu, Wei-Guo Chen, Li-Ping Jiang, Jie Chen, Zhao-Peng Hu, Yong-Jian Huang, Zhi-Wei Zhou, Gui-Liang Wang, Hao Lin, Shu-Feng Zhou

**Affiliations:** 1 Department of Gastroenterology, the Affiliated Pingxiang Hospital of Southern Medical University, Pingxiang, Jiangxi, China; 2 Animal Laboratory, the Affiliated Pingxiang Hospital of Southern Medical University, Pingxiang, Jiangxi, China; 3 Department of Pharmacology, the Affiliated Pingxiang Hospital of Southern Medical University, Pingxiang, Jiangxi, China; 4 Department of Pathology, the Affiliated Pingxiang Hospital of Southern Medical University, Pingxiang, Jiangxi, China; 5 Department of Clinical Laboratory, the Affiliated Pingxiang Hospital of Southern Medical University, Pingxiang, Jiangxi, China; 6 Department of Pharmaceutical Science, College of Pharmacy, University of South Florida, Tampa, Florida, United States of America; University of Valencia, Spain

## Abstract

Accumulating evidence suggests that intestinal epithelial barrier dysfunction plays an important role in the pathogenesis of hepatic cirrhosis and its complications such as gastrointestinal injury and hepatic encephalopathy. To date, there is no cure for cirrhosis-associated intestinal mucosal lesion and ulcer. This study aimed to investigate the effect of oxymatrine on intestinal epithelial barrier function and the underlying mechanism in carbon tetrachloride (CCl_4_)-induced cirrhotic rats. Thirty CCl_4_-induced cirrhotic rats were randomly divided into treatment group, which received oxymatrine treatment (63 mg/kg), and non-treatment group, which received the same dose of 5% glucose solution (vehicle). The blank group (n = 10 healthy rats) received no treatment. Terminal ileal samples were collected for histopathological examination. The expression level of nuclear factor-κB (NF-κB) p65 in ileal tissue was evaluated by immunohistochemistry. The gene and protein expression levels of tumor necrosis factor-α (TNF-α) and interleukin 6 (IL-6) in ileal tissues were analyzed by reverse-transcriptase polymerase chain reaction (RT-PCR) and enzyme-linked immunosorbent assay (ELISA), respectively. Additionally, plasma endotoxin level was determined. In comparison to the blank group, a significant alteration in the morphology of intestinal mucosal villi in the non-treatment group was observed. The intestinal mucosal villi were atrophic, shorter, and fractured, and inflammatory cells were infiltrated into the lamina propria and muscular layer. Besides, serious swell of villi and loose structure of mucous membrane were observed. Oxymatrine reversed the CCl_4_-induced histological changes and restored intestinal barrier integrity. Moreover, oxymatrine reduced the protein expression level of NF-κB p65, TNF-α, and IL-6, which were elevated in the vehicle-treated group. In addition, the serum endotoxin level was significantly decreased after oxymatrine treatment in CCl_4_-induced cirrhotic rats. The results indicate that oxymatrine improves intestinal barrier function via NF-κB-mediated signaling pathway and may be used as a new protecting agent for cirrhosis-associated intestinal mucosal damage.

## Introduction

Cirrhosis is the advanced stage of liver fibrosis and a major risk factor of hepatocellular carcinoma. Cirrhosis is a common disease-related cause of hospitalization and death in the United States (US). The prevalence of cirrhosis is about 0.15% in the US and there are more than 31,000 deaths each year resulting from cirrhosis [1]. There is evidence that bacterial translocation (BT) from the intestinal lumen to mesenteric lymph nodes or other extra intestinal locations is an important contributing factor to the pathogenesis of cirrhosis and its complications such as gastrointestinal injury and hepatic encephalopathy. Clinical studies have documented that 25–30% of cirrhotic patients have BT [2]. Intestinal epithelial barrier has an important role in the regulation of water and ion fluxes, nutrient absorption and host protection and integrity of intestinal epithelial barrier is essential for maintaining its physiological functions [1]. Under pathological conditions, disruption of intestinal epithelial barrier integrity leads to intestinal epithelial barrier dysfunction [3] which facilitates BT and consequently results in remarkable inflammatory responses and eventually tissue injuries [4].

Inflammation response is a crucial part of the defense mechanisms against bacteria and bacterial product-induced tissue damages [5], [6], and it has been implicated in the initiation, development, and progression of intestinal barrier dysfunction, BT, and eventually cirrhosis. Nuclear factor κB (NF-κB) family comprises of RelA, c-Rel, RelB, and NF-κB1(p105/p50) and they are critical transcription factors involved in various cellular responses to stimuli such as cytokines and bacterial/viral antigens [7]–[9]. In particular, NF-κB plays a pivotal role in the initiation and regulation of inflammatory and immune responses by interplaying with various signaling pathways, which regulates the intracellular and extracellular level of pro-inflammatory cytokines, such as interferon (IFN)-γ, tumor necrosis factor (TNF)-α, interleukin (IL)-1β, IL-6, and IL-13 [9]–[11]. On the other hand, intestinal barrier dysfunction leads to intestinal inflammation and causes the release of various pro-inflammatory cytokines, consequently increasing the level of cytokines and then activating the NF-κB signaling pathway. This will in turn enhance the recruitment of inflammatory cells and trigger the production of more pro-inflammatory cytokines [12]. Furthermore, these cytokines often exhibit synergistic effects on inflammatory response and induce the production of secondary mediators such as chemokines, prostaglandins, and platelet-activating factors [13], resulting in aggravated inflammation and intestinal barrier injury. Therefore, inhibition of NF-κB p65 to decrease the release of the cytokines may be a potential strategy in the control of intestinal inflammation and may be one of the effective approaches in preventing the damage of intestinal barrier in clinical practice.

**Figure 1:**
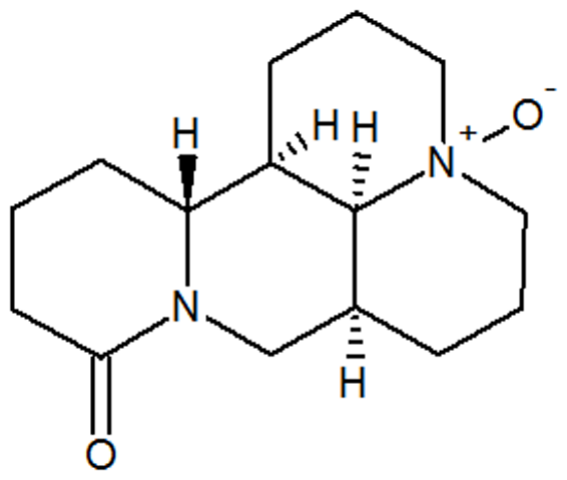
The chemical structure of oxymatrine. It is a quinolizidine alkaloid derived from the traditional Chinese herb Radix *Sophora flavescens* (Ku Shen in Chinese).

Oxymatrine (**Figure 1**), a quinolizidine alkaloid derived from traditional Chinese herb Radix *Sophora flavescens* (??, Ku Shen in Chinese), has a wide range of preclinical pharmacological activities, including anti-oxidative, anti-viral, anti-bacterial, hepatoprotective, and immune-modulating activities [14]–[16]. In clinical settings, oxymatrine has been primarily used for the treatment of liver diseases, due to its purported anti-viral and anti-inflammatory effects. Several preclinical studies have evaluated its beneficial effects and investigated the underlying mechanism. Shi *et al* [17], [18] showed that oxymatrine simultaneously down-regulated sterol regulatory element binding transcription factor 1 and up-regulated peroxisome proliferator activated receptor alpha mediated metabolic pathways to attenuate hepatic steatosis in rats with non-alcoholic fatty liver disease. It has been reported that oxymatrine protected animals against ischemia and reperfusion-induced liver, heart, and intestinal injuries involving extracellular signal regulated kinase, c-Jun N-terminal kinase, and p38 mitogen-activated protein kinases signaling pathways [19]–[21]. In addition, the inhibitory effect of oxymatrine on NF-κB signaling pathway has been reported in pancreatic cancer [22] and cerebral ischemia in animals [23]. However, the effect of oxymatrine on NF-κB signaling pathway and the association with intestinal epithelial barrier dysfunction is unclear. As such, we hypothesize that oxymatrine can restore the integrity and function of intestinal epithelial barrier via suppressing the activation of NF-κB signaling pathway. To test this hypothesis, we have investigated the effect of oxymatrine on intestinal epithelial barrier function and the underlying mechanism in CCl_4_-induced liver cirrhotic rats.

## Materials and Methods

### Animals

Fifty male Sprague-Dawley rats (180 to 220 g) were purchased from Shanghai SLAC Laboratory Animal Co. Ltd. (Shanghai, China). All animals were housed in plastic cages containing wood shaving and maintained in a room at 22–25°C with a 12-hr light/night cycle with free access to standard laboratory diet and water. The animal study protocol was approved by the Ethics Committee for Animal Experiments of Southern Medical University Pingxiang Hospital, Pingxiang, Jiangxi, China.

### Experimental protocol

The rats were randomly divided into the blank group with healthy rats, cirrhotic rat group with vehicle treatment only, and cirrhotic rat group with oxymatrine treatment (n = 10–15). The animals' weights were monitored prior to the start of experiment and then checked 2 times per week (Monday and Thursday) during the period of experiment. In the cirrhotic group, animals were subcutaneous injected 40% CCl_4_ in a 2∶3 mixture with olive oil (0.3 mL/kg), twice a week for 12 weeks, as described previously [24], to induce cirrhosis model. Liver cirrhosis was established in 30 rats at the end of 12 wks. The 30 rats treated with CCl_4_ were further divided into non-treatment group (n = 15) and treatment group (n = 15). Rats in the blank group and vehicle-treated group received intramuscular injection with 5% glucose solution, once a day for 4 weeks. Rats in the treatment group received intramuscular injection with oxymatrine (63 mg/kg) dissolved in a 5% glucose solution, once a day for 4 weeks. 40% CCl_4_ was continued during the experimental period of 16 weeks. At the end of the 16 week-long experimental period, all rats were anesthetized by injecting 3% chloral hydrate one day after the last dose of administration. Animals will be euthanized, if the weight drops 10%. The rats will be immediately euthanized if other symptoms such as pale mucous membranes, shivering, failure to respond to stimuli, piloerection, matted hair coat, soiled anogenital/vent area and vocalization are observed. Due to occurrence of the above mentioned symptoms, there were 10 rats euthanized during the period of experiment. Tissue samples were collected and blood samples were collected from portal vein, all samples were stored in −80°C freezer for further analysis.

### Histopathological study

The terminal ileal samples were collected for routine histopathological examination. The ileal tissue was tiled on a sterile virgin paper to evaluate the degree of intestinal injury after flushed with 0.9% saline water. The mucosal damage was evaluated using microscopic imaging. Sections of 4 µm-thick were stained with hematoxylin and eosin (HE) and observed under light microscopy (Nikon Eclipse 50i; Kanagawa, Japan).

### Evaluation of the concentration of TNF-α and IL-6 in ileal tissue by ELISA

Part of the frozen ileal tissue (5 mg) was cut into pieces and homogenized in the proteinase inhibitors containing pre-cooled phosphate buffered saline (PBS) solution. The supernatant of the homogenate was collected to evaluate the concentrations of TNF-α and IL-6 by ELISA using TNF-α and IL-6 ELISA kits (Shanghai BlueGene Biotech CO., LTD. China) according to the manufacturer's instructions. The concentration of TNF- α and IL-6 were determined via comparing the absorbance of the samples to the standard curves and normalized by the concentrations of protein.

### RNA isolation and reverse transcriptase polymerase chain reaction (RT-PCR)

The mRNA expression level of *TNF-α* and *IL-6* was assessed using RT-PCR and normalized by *GAPDH*. Briefly, total RNA from ileal tissue was extracted using Trizol reagent (Sigma, St. Louis, MO, USA) by the double-step method, and reverse transcribed into cDNA. The reaction was started at 42°C for 60 min and terminated by placing it on ice after deactivation at 70°C for 10 min. The resultant cDNA was used for subsequent PCR. The primer sequences used for detecting *TNF-α*, *IL-6*, and *GAPDH* were shown in **Table 1**. Amplification was performed in 35 cycles, including denaturation for 30 s at 95°C, annealing for 45 s at 55°C or 57°C, and extension for 60 s at 72°C. The products were separated in 1% agarose gel with GoldenView staining and quantitated using a digital camera and an image analysis system (Vilber Lourmat, Marne La Vallée, France).

**Table 1:** Forward and reverse primers used in reverse transcription polymerase chain reaction.

Rat gene	Primer sequence (5′-3′)	Annealing temperature (°C)	Product size (bp)
*GAPDH*	5′-CAGTGCCAGCCTCGTCTCAT-3′ 5′-AGGGGCCATCCACAGTCTTC-3′	58	595
*TNF-α*	5′-CACCACGCTCTTCTGTCTACTG-3′ 5′-AGATAAGGTACAGCCCATCTGC-3′	57	281
*IL-6*	5′-CAAGAGACTTCCAGCCAGTTG-3′ 5′-GAAACGGAACTCCAGAAGACC-3′	55	350

TNF-α: tumor necrosis factor-α; IL: interleukin.

### Detection of the expression level of NF-κB p65 in ileal tissue

The expression level of NF-κB p65 in ileal tissue was assessed by immunohistochemistry. Briefly, the sections were firstly de-paraffinized in xylene and rehydrated in a descending ethanol series. After de-waxing and rehydration, antigen was retrieved by microwave for 15 min and endogenous peroxidase activity was blocked by 3% H_2_O_2_ in methanol at room temperature for 20 min. The sections were probed with specific polyclonal rabbit anti-rat NF-κB p65 serum (Santa Cruz Biotechnology, Inc, Santa Cruz, CA, USA) for 12 hr at 4°C. Negative control was incubated with normal rabbit serum under the same conditions. After incubation with the primary antibody, the slides were washed by PBS and incubated with respective secondary antibody and 0.1% diaminobenzidine substrate. Ten fields were randomly selected (400× magnification) and the results were quantitated.

### Measurement of serum endotoxin level

Endotoxin quantification was carried out using a Kinetic Turbidimetric LAL Kit (Xiamen Limulus Experimental Reagents Factory, Xiamen, China) according the manufacturer's instructions. Briefly, venous blood was collected and centrifuged at 1,000× *g* for 1 min, and then it was transferred into 100 µl sample solution. Samples were incubated at 70°C for 10 min, following ice-cold water incubation for 3 min. Then, limulus reagents were added into 200 µl processed mixing solution, and 100 µl of mixture was transferred to a microplate (96-well) for analysis. A standard curve was used to calculate the concentration of endotoxin. The optical density of reaction solution was determined for the calculation of endotoxin concentration (EU/ml).

### Statistical analysis

SPSS 16.0 (IBM Inc., Chicago, IL, USA) was used for the statistical analysis. Data are expressed as mean ±SD. One-way analysis of variance (ANOVA) was used to evaluate differences in the concentration of TNF-α and IL-6, ratio of NF-κB p65 positive cells, and the expression of mRNA in ileal tissue. The level of endotoxin was analyzed by Kruskal-Wallis *H* test. *P*<0.05 was considered to be statistically significant.

## Results

### Oxymatrine improves CCl_4_-induced histological changes in ileal tissue

**Figure 2:**
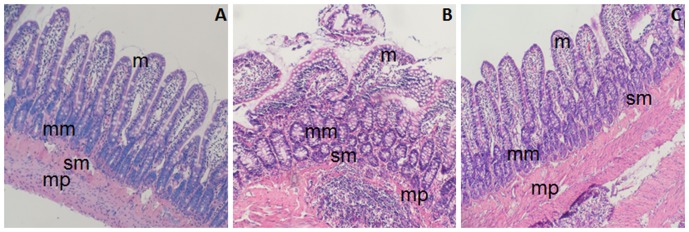
Hematoxylin and eosin staining of rat ileal tissue (HE, ×200) from different groups. A: blank (normal) group; B: CCl_4_-treated rats receiving the vehicle only; and C: oxymatrine treatment group. m: mucosa, mm: muscularis mucosae, mp: muscularis proparia, and sm: submucosa.

The ileum is a terminal section of the small intestine and forms the connection to the large intestine. The architecture of ileal tissue reflects the pathophysiologic alteration of intestinal tissue in response to the stimuli [25]. Cirrhosis is often accompanied by BT due to the disruption of intestinal epithelial barrier. As such, we examined the effect of oxymatrine on morphological alterations in intestinal epithelial barrier. **Figure 2**A showed the typical architecture of normal ileal tissue including mucosa (m), muscularis mucosae (mm), muscularis proparia (mp), and submucosa (sm). As shown in **Figure 2**A, the architecture of mucosa under light microscopy was intact in healthy rats. The villi were present as finger-like projections and well arranged in intestinal tissue and the infiltration of inflammatory cells was hardly observed in the intestinal mucosa. Muscularis mucosae was intact at the boundary of the mucosa and submucosa in healthy rats (**Figure 2**A). The structure of glandular epithelia was typically cylindrical. In CCl_4_-induced cirrhotic rats, massive destruction of the intestinal mucosa was observed with atrophic, shorter, and fractured villi and infiltration of inflammatory cells into the lamina propria and muscular layer. The cylindrical shapes of glandular epithelia disappeared and showed as irregular structures. Besides, serious swell of villi and loose structure of mucous membrane were observed in the non-treatment group (**Figure 2**B). In contrast, oxymatrine ameliorated CCl_4_-induced tissue damages in ileal tissue (**Figure 2**C). The order of intestinal mucosal villi was restored with slight edema and improved integrity of mucous membrane was observed. The cylindrical structure of glandular epithelia was restored by oxymatrine treatment. Moreover, oxymatrine significantly decreased CCl_4_-induced infiltration of inflammatory cells (**Figure 2**C). These results demonstrate that oxymatrine restores the integrity and function of intestinal epithelial barrier and significantly ameliorate CCl_4_-induced intestinal epithelial barrier damages.

### Oxymatrine suppresses CCl_4_-induced release of TNF-α and IL-6 in small intestine

**Figure 3:**
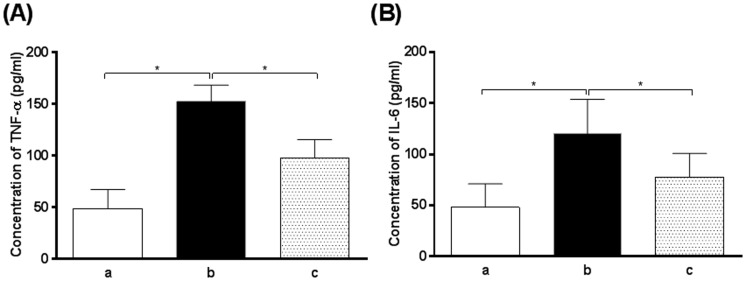
The concentrations of TNF-α and IL-6 in rat ileal tissues from different groups. a: blank (normal) group; b: CCl_4_-treated rats receiving the vehicle only and c: oxymatrine treatment group. (A): The concentration of TNF-α in rat ileal tissues and (B): the concentration of IL-6 in rat ileal tissues. *P<0.01 by one-way ANOVA.

**Figure 4:**
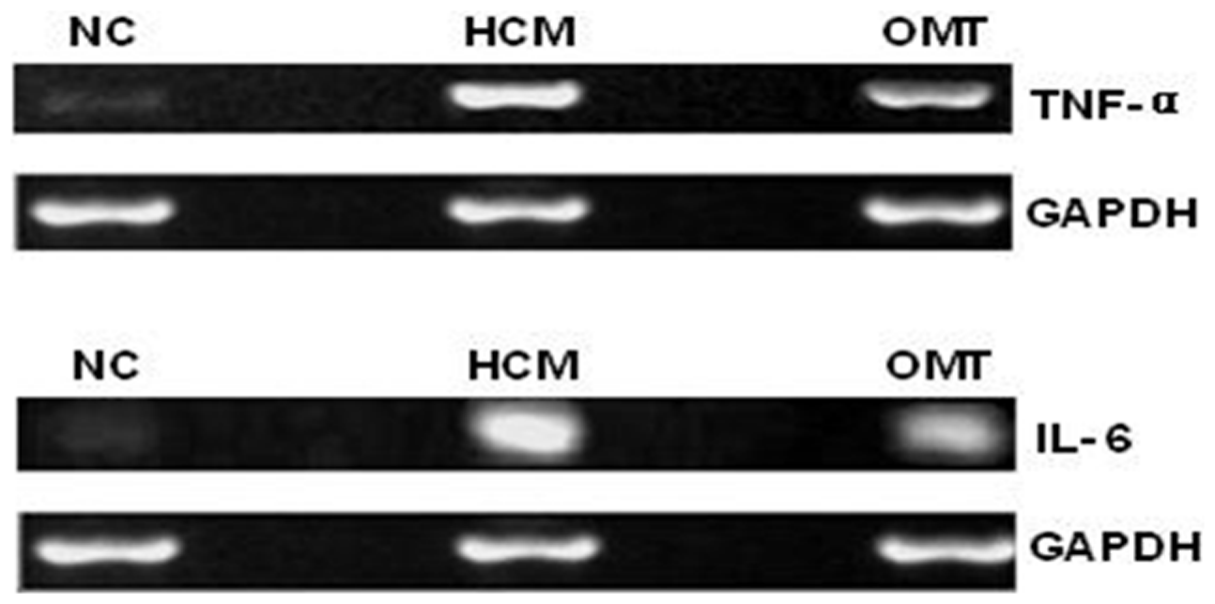
Representative bands for *TNF-α* and *IL-6* mRNAs in rat ileal tissues from different groups. RT-PCR was used to determine the levels of *TNF-α* and *IL-6* mRNAs. *GAPDH* was applied as the control. Lane 1: blank (normal) group; Lane 2: CCl_4_-treated rats receiving the vehicle only group; and Lane 3: oxymatrine treatment group. TNF-α: tumor necrosis factor-α; IL-6: interleukin-6.

TNF-α and IL-6 are two major pro-inflammatory cytokines released in intestinal mucosa during the disruption of intestinal epithelial barrier integrity, which leads to intestinal epithelial barrier dysfunction [4]. In order to examine the effect of oxymatrine on inflammatory response, the expression level of intestinal TNF-α and IL-6 was determined. In comparison to the blank group (healthy rats), there was a 3.1- and 2.3-fold increase in the expression level of TNF-α and IL-6 observed in the vehicle-treated group, respectively (*P*<0.01). Oxymatrine significantly reduced the expression level of TNF-α and IL-6 by 35.9% and 34.3%, respectively, compared to non-treatment group (*P*<0.01) (**Figure 3**). Following the examination of the expression level of TNF-α and IL-6, the transcriptional levels of *TNF-α* and *IL-6* were also evaluated. The mRNA expression levels of *TNF-α* and *IL-6* in non-treatment group were significantly higher than that in the blank (normal) group. Oxymatrine significantly decreased the mRNA expression level of *TNF-α* and *IL-6* compared to the non-treatment group (**Figure 4**). The results show that oxymatrine has potent anti-inflammatory effect which may, at least in part, explain the beneficial action of oxymatrine on the restoration of the integrity of intestinal epithelial barrier in cirrhotic rats.

### Oxymatrine inhibits CCl_4_-induced activation of NF-κB p65 in rat ileal tissue

**Figure 5:**
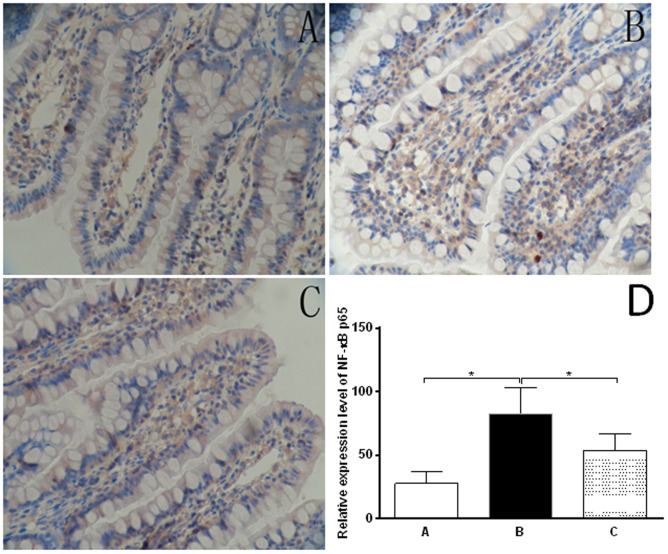
The immunohistochemical observation of stained NF-κB p65 in rat ileal tissues from different groups (SP×400). The ileal sections were firstly de-paraffinized in xylene and rehydrated and antigen was retrieved by microwave and endogenous peroxidase activity was blocked by 3% H_2_O_2_ in methanol. The sections were probed with specific polyclonal rabbit anti-rat NF-κB p65 serum and the slides were then washed with PBS and incubated with respective secondary antibody and 0.1% diaminobenzidine substrate. Ten fields were randomly selected (400× magnification) and the results were quantitated. A: blank (normal) group; B: CCl_4_-treated rats receiving the vehicle only group; C: oxymatrine treatment group; and D: Bar graph showing the relative expression level of NF-κB p65 in rat ileal tissues. *P<0.01 by one-way ANOVA.

NF-κB is a critical transcriptional factor regulating the expression of a number of genes involving inflammatory response [7]. Since we have observed the inhibitory effect of oxymatrine on both transcriptional and posttranscriptional level of TNF-α and IL-6, we further investigated the effect of oxymatrine on upstream modulators to elucidate the underlying mechanism of the beneficial actions of oxymatrine on the inflammatory response. The expression and location of NF-κB p65 was examined by immunohistochemistry in ileal tissue. NF-κB p65 was mainly expressed in the nucleus, cytoplasm of epithelia and macrophages (**Figure 5**A). The expression level of NF-κB p65 in non-treatment group was significantly higher than that in the blank group (78.7±8.5% vs 28.1±9.9%) (**Figure 5**B). Oxymatrine significantly reduced the expression level of NF-κB compared to the cirrhotic rats without oxymatrine treatment (52.2%±11.0% vs 78.7%±8.5%) (**Figure 5**C). The results indicate that oxymatrine suppresses the inflammation response in CCl_4_-induced cirrhotic rats via NF-κB signaling pathway.

### Oxymatrine reduces CCl_4_-induced plasma endotoxin level

Endotoxin, a component of cell wall of gram negative bacteria, can initiate inflammatory response and induce the production of pro-inflammatory cytokines such as TNF-α and IL-6 [26]. To further determine the beneficial effect of oxymatrine on inflammation, the level of plasma endotoxin was evaluated. As shown in **Table 2**, the level of plasma endotoxin was significantly increased in the non-treatment group in comparison to normal rats (*P*<0.01). Oxymatrine remarkably decreased CCl_4_-induced plasma endotoxin production compared to the non-treatment group (*P*<0.01). The reducing effect on plasma endotoxin level of oxymatrine may contribute to its beneficial action on the restoration of intestinal epithelial barrier function.

**Table 2:** Distribution of endotoxin levels in serum of different groups.

Group	n	0∼0.035 (EU/ml)	0.035∼0.1 (EU/ml)	0.1∼1.0 (EU/ml)	>1.0 (EU/ml)	Mean rank
Blank group	10	10	0	0	0	10.50^a^
Vehicle-treated group	15	2	4	7	2	28.90
Oxymatrine-treated group	15	8	5	1	1	18.77^a^^,^ b

a *P*<0.01 vs non-treatment group;

b *P*<0.05 vs blank (normal) group.

## Discussion

Intestinal epithelial barrier dysfunction is an important contributor facilitating the BT which has been involved in the pathogenesis of cirrhosis and its complications. Better understanding of the role and underlying mechanism for intestinal epithelial barrier dysfunction will provide further insights into the pathogenesis and progression of cirrhosis and identification of new efficacious therapeutic interventions. In the present study, we examined the effect of oxymatrine on CCl_4_-induced liver cirrhosis in rats. The results showed that oxymatrine restored the integrity and function of intestinal epithelial barrier in CCl_4_- induced liver cirrhotic rats. These benefit effects can be ascribed, at least in part, to the inhibitory effect of oxymatrine on NF-κB signaling pathway.

The small and large intestines share certain histologic characteristics. The intestinal epithelial barrier mainly consists of epithelial and immunological barriers. The disruption of intestinal epithelial barrier is featured with significant morphological changes in the architecture and integrity of the barrier, such as loose of junction complex, infiltration and invasion of immune cell, which result in intestinal epithelial barrier dysfunction [4], [27]. In the present study, we examined the architecture of ileal tissue, which is the terminal part of the small intestine and connects the large intestine. The architecture alteration of ileal tissue can reflect the response of the intestine to the stimuli. Our findings showed that oxymatrine remarkably restored the disrupted architecture of intestinal epithelial barrier in CCl_4_-induced cirrhosis rats. On the other hand, it has been demonstrated that dysfunction of intestinal epithelial barrier favors BT. Accumulating evidence suggests that translocation of bacterial and bacterial products plays a causal role in the pathogenesis of chronic liver diseases and their complications [28]. In agreement with previous studies [6], [29], our findings showed significant morphological alterations in intestinal epithelial barrier in CCl_4_-induced liver cirrhotic rats. The results indicate that disruption of intestinal epithelial barrier is involved in the pathogenesis of liver cirrhosis in rats and oxymatrine can restore the integrity of intestinal epithelial barrier.

Systemic inflammation is a major characteristic of cirrhosis and other chronic liver diseases with increased levels of pro-inflammatory cytokines and chemokines such as TNF-α and IL-6 [4], [5], which in turn can disrupt the integrity and function of the intestinal epithelial barrier. We have observed a significant elevation in both gene and protein expression level of TNF-α and IL-6 in cirrhotic rats and a significant increase in the expression of NF-κB p65 in cirrhotic rats compared with the normal group. Furthermore, the immunohistochemistry results showed a correlation between the expression level of TNF-α and IL-6 and the disruption of intestinal epithelial barrier. It indicates the role of inflammation in the intestinal epithelial barrier dysfunction involving NF-κB p65 signaling pathway. Indeed, NF-κB is a transcription factor which regulates the expression of a number of genes encoding pro-inflammatory cytokines, their corresponding receptors, and associate signaling molecules [7]–[9].

In previous studies, oxymatrine has been reported to be effective in the treatment of colitis, mainly by regulating the NF-κB signaling pathway and reducing the production of inflammatory cytokines. For example, it has been demonstrated that oxymatrine inhibits NF-κB p65 nuclear translocation and ameliorates intestinal inflammation [15], [17], [22], [30], [31]. Therefore, the pharmacological effects of oxymatrine may be ascribed to the inhibition of NF-κB activation and the decrease in cytokines expression. It has also been demonstrated that cytokine blockade with anti-TNF-α monoclonal antibody can decrease the incidence of BT in cirrhotic rats with ascites [32], and the integrity of intestinal barrier can be preserved in the IL-6 knockout mice [33]. In addition, Yang *et al* [24] showed that salvianolate inhibited the expression of TNF-α and IL-6 and ameliorated intestinal mucosal injury of cirrhotic rats. In agreement with previous studies, we observed that expression level of NF-κB p65, TNF-α, and IL-6 was significantly reduced in animals that received oxymatrine treatment. Among the pro-inflammatory mediators, TNF-α has a significant role in the initiation, development and worsening of intestinal barrier dysfunction [34], [35]. Previous studies have shown that TNF-α is an important mediator of bacterial invasion [36], and it can initiate the production of cytokines such as IL-6 and IFN-γ, which can exacerbate the intestinal epithelial barrier injury [37]. In clinic settings, the serum level of TNF-α and IL-6 are significantly increased in cirrhotic patients [38]. In this study, the altered intestinal epithelial barrier function in rats with liver cirrhosis was observed with an increase in TNF-α and IL-6 expression levels in small intestine. These observations suggest that oxymatrine may directly inhibit inflammation response contributing to the restoration of disrupted intestinal epithelial barrier function in cirrhotic rats. Therefore, suppressing the activation of NF-κB signaling pathway and blocking the inflammation response may present new therapeutic strategies for the intestinal epithelial barrier injury.

Additionally, in order to further evaluate the improvement of intestinal mucosal barrier function after the treatment of oxymatrine, we detected the plasma level of endotoxin. It has been reported that endotoxin can activate toll-like receptor 4, initiate inflammatory response, stimulate macrophages and monocytes, and enhance the production of pro-inflammatory cytokines such as TNF-α and IL-6 [26], [39]. In our study, the plasma endotoxin level was significantly increased in non-treatment group. However, in agreement with the improvement of intestinal histopathological changes in the treatment group, plasma endotoxin level was significantly reduced in rats after treatment with oxymatrine. These results suggest that intestinal epithelial barrier injury could increase intestinal permeability and result in an increase in plasma endotoxin level, which may, in turn, further aggravate intestinal epithelial barrier injury. Therefore, decreasing endotoxin level may block this vicious circle. These beneficial effects of oxymatrine are ascribed, at least in part, to the inhibition of NF-κB signaling pathway and resultant attenuation of the production of pro-inflammatory cytokines, thereby improving intestinal epithelial barrier function.

In the present study, there are several limitations, including the assessment of the function for the intestinal epithelial barrier and the identification of inflammatory cells. Although we have observed the remarkable ameliorating effect of oxymatrine on architecture of intestinal epithelial barrier in cirrhotic rats and the results can indicate the functional improvement of intestinal epithelial barrier, the functional experiments are important and can directly approve the beneficial effect of oxymatrine. The identification of inflammatory cells from ileal tissue is another limitation. In order to identify the role of inflammation response in the BT and intestinal epithelial barrier dysfunction, the classification for the type of inflammatory cells in ileal tissue is important.

In conclusion, oxymatrine has shown significant protecting effect on the intestinal epithelia in CCl_4_-induced cirrhotic rats. The beneficial effect is mainly ascribed to its inhibitory activities on production of local pro-inflammatory cytokines and endotoxin, probably via suppression of NF-κB-mediated signaling pathway. Oxymatrine may represent a new type of therapeutic agent used to protect from cirrhosis-associated intestinal damages. Further mechanistic studies are needed to explore the molecular targets of oxymatrine in various tissues.
